# Combination therapy only shows short-term superiority over monotherapy on ureteral stent-related symptoms – outcome from a randomized controlled trial

**DOI:** 10.1186/s12894-016-0186-y

**Published:** 2016-11-15

**Authors:** Qinyu Liu, Banghua Liao, Ruochen Zhang, Tao Jin, Liang Zhou, Deyi Luo, Jiaming Liu, Hong Li, Kunjie Wang

**Affiliations:** Department of Urology, Institute of Urology (Laboratory of Reconstructive Urology), West China Hospital, Sichuan University, Chengdu, 610041 Sichuan People’s Republic of China

**Keywords:** Stent-related symptoms, Medication therapy management, Muscarinic antagonists, Adrenergic alpha-1 receptor antagonists

## Abstract

**Background:**

Controversy remains on the superiority of combination therapy over monotherapy on ureteral stent-related symptoms (SRSs). We tend to explore if there is a necessity of combination therapy.

**Methods:**

One hundred cases of unilateral upper urinary tract calculi with stent insertion (pre and post flexible ureteroscopy) were randomized into 4 groups, given non-treatment, solifenacin, tamsulosin or combination respectively. Eight times of follow-ups were given after each insertion.

**Results:**

SRSs released spontaneously within 4 days after insertion (*p* = 0.017) but then stay with no further improvement. Benefit of solifenacin on flank pain started showing after day4 (*p* = 0.002), which was comparable to that of tamsulosin and combination (*p* = 0.914 vs 0.195). Combination therapy showed superiority over both monotherapy before day4, but after then solifenacin and tamsulosin showed similar effectiveness with the combination therapy on both bladder pain (*p* = 0.229 vs 0.394) and urgency (*p* = 0.813 vs 0.974). No improvement on hematuria or frequency was observed in each group.

**Conclusions:**

Combination therapy takes effect faster but shows no supervisory after the first few days compared with monotherapy.

**Trial registration:**

The study protocol was registered on Chinese Clinical Trial Register on April 17th, 2013 (registration number: ChiCTR-TRC-13003148).

**Electronic supplementary material:**

The online version of this article (doi:10.1186/s12894-016-0186-y) contains supplementary material, which is available to authorized users.

## Background

A vast majority of patients with indwelling ureteral stent are suffering from stent-related symptoms (SRSs) with poor quality of life (QoL), and storage symptoms and body pain are the most troublesome [1, 2]. Currently it is hypothesized that bladder discomfort, lower urinary tract symptoms (LUTS) and hematuria are due to mechanical irritation of bladder trigone as well as bladder neck, while flank pain is associated with vesicoureteric reflux and evidences showed antireflux stent can minimize the pain [3]. As a consequence, efforts such as improving stent design and composition and investigating medical therapy have been made to solve this problem [4–6]. So far many researches have shown that α-blockers and anticholinergic agents both can ease these discomforts and ultimately improve the QoL [7] . However, there’re still not many researches on comparison between monotherapy and combination. In addition, some most recent published papers made different voices: while former researches with International Prostate Symptom Score (IPSS) found combination therapies provided preferable outcomes, some most current ones declared that monotherapies functioned equally with the combination in Ureteric Stent Symptom Questionnaire (USSQ) assessment [8, 9].

Basing on the background above, we conducted a randomized controlled trial to evaluate the efficacy of solifenacin, tamsulosin and the combination therapy, and meanwhile to explore SRSs’ development features with time as secondary outcomes.

## Methods

### Subjects and treatments

An open-label, randomized, controlled study was conducted at West China Hospital of Sichuan University from Feb 2014 to May 2015. Inclusion criteria were as followed: (1) aged 18–60 years with unilateral nephrolithiasis ≤2 cm; (2) 4.7Fr ureteral stent being inserted before and after flexible ureteroscopic lithotripsy. The exclusion criteria included: (1) a history of urinary tract surgery; (2) a history of LUTS related to benign prostatic hyperplasia or infection; (3) concomitant use of other antiadrenergics, anticholinergics, and analgesics; (4) a history of neurogenic bladder, overactive bladder syndrome, neurologic and psychiatric diseases, chronic prostatitis and urinary tract abnormalities; (5) drug allergy; (6) having major complications after the surgery.

4.7Fr ureteral stents (INLAY®, Bard Inc.) of 26 cm were inserted in all cases through cystoscopy 2 weeks before the ureteroscopic surgery. A stent of the same size was inserted after lithotripsy under general anesthesia within the flexible ureteroscopic surgery. X-ray plain films were done after both insertions to make sure the stents were in correct position since inappropriate stent location would worsen LUTS and affect the QoL severely [10, 11]. Patients were told to drink more than 2500 ml water per day and avoid aggravating physical activities after insertion. Patients were discharged on the third day following lithotripsy surgery.

### Randomization, follow-up, assessment of outcomes

Patients were randomized into one of four groups, namely C (control), S (solifenacin 5 mg once daily), T (tamsulosin 0.2 mg once daily), and S + T (solifenacin and tamsulosin combination).

Follow-ups were performed on day 1, 2, 3, 4, 5, 6, 10, and 14 after stent insertion on phone. Questions on urinary symptoms were selected from USSQ to assess bladder irritation, while a visual analogue scale (VAS) and a seven-score QoL scale were adopted for body pain and QoL assessment. Every patient had two series of follow-ups (pre- and post-lithotripsy) as self-control. Data of patients who missed more than twice dose or follow-ups throughout the follow-up duration were excluded in the final analysis. Also a questionnaire aiming at adverse events was taken on day14 to evaluate the safety.

The primary outcome was the urinary symptom score of the given questionnaire. The secondary outcomes included scores in every single symptom assumed in the current study, the score of quality of life and initial effect time.

### Sample size and statistical analysis

Sample size was calculated with the standard deviation of the urinary symptom domain of 4 as observed in our preliminary test of patients given no treatment and the following assumed post-stent urinary symptom scores: no treatment (14), tamsulosin (11), solifenacin (11) tamsulosin, and solifenacin (10). For α = 0.05 and β = 0.1, the minimal sample size needed for each group was 20. Assuming a 20% withdrawal rate, we decided to have 25 as the least sample size needed for each group and recruit as many as possible during the research period.

SPSS 20.0 was used for statistical analysis. Repeated measures analysis of variance was used to compare variables between groups. Chi-square and ANOVA tests were used to compare ratios and mean values between groups or different follow-up days. Logistic regression was used to reveal relevance between variables. A *p*-value < 0.05 indicated statistically significant differences in the current study.

## Results

**Fig. 1:**
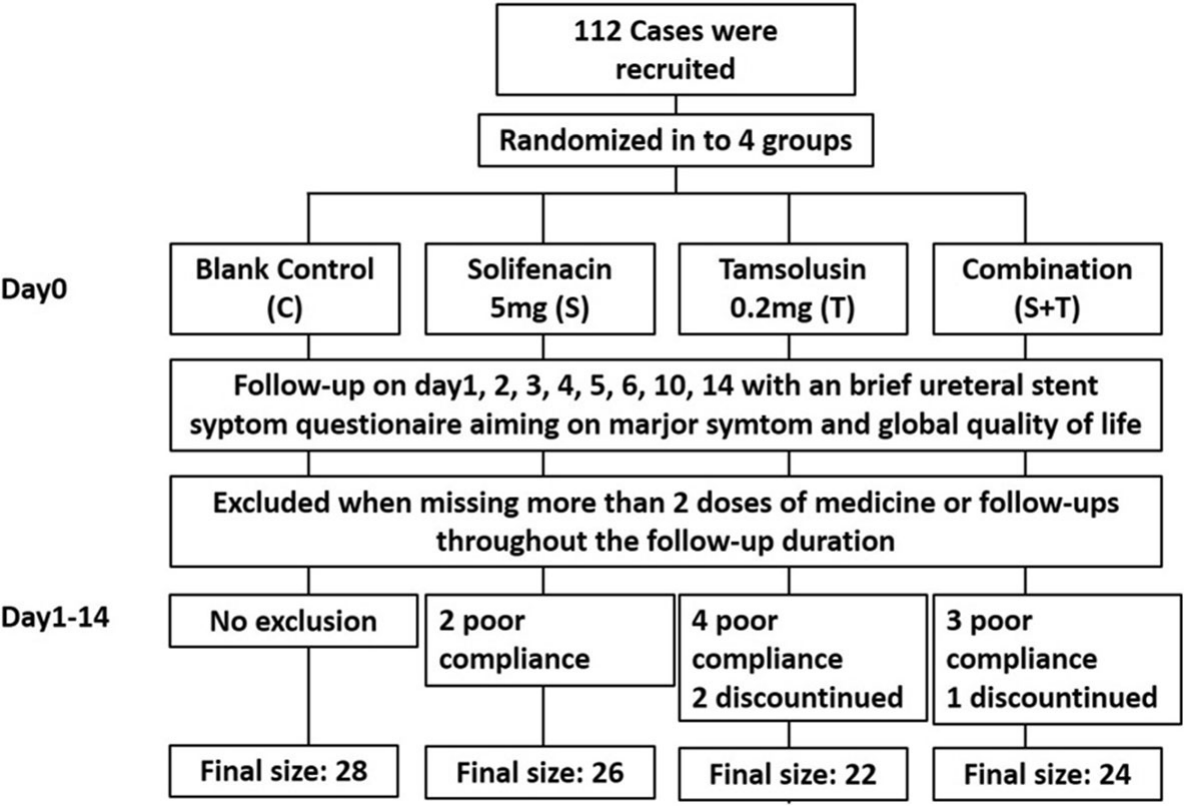
Flow diagram of the current study

Finally, a total of 112 cases were recruited. With 12 cases (10.71%) not appropriate for final analysis due to loss of follow-up or poor compliance, the final sample size was 100 (group C 28, S 26, T 22 and S + T 24, Fig. 1). No significant differences showed in age, height, weight or gender among the 4 groups (*p* = 0.633, 0.131, 0.674, 0.337) (Table 1). None of participants were found with a history of urinary tract surgery, LUTS related to benign prostatic hyperplasia or infection, concomitant use of other similar drugs or any other comorbidities that may confuse the assessment of SRSs.

**Table 1:** Popularity characteristic of the current study

Variables	Group c	Group s	Group t	Group s+t
Number of cases	28	26	22	24
Gender, n (%)				
Male	20 (71.4)	20 (76.9)	12 (54.5)	18 (75.0)
Female	8 (28.6)	6 (23.1)	10 (45.5)	6 (25.0)
Age (year), mean ± SD	40.00 ± 8.24	41.55 ± 10.63	43.1 ± 12.10	44.00 ± 12.16
Height (cm), mean ± SD	165.75 ± 7.92	167.67 ± 6.05	165.30 ± 8.03	162.44 ± 5.42
Weight (kg), mean ± SD	67.08 ± 12.33	64.83 ± 10.80	67.10 ± 14.71	63.00 ± 11.24

*P* < 0.05 for age, height, weight and gender among the 4 groups

### Characteristics of SRSs in the control group

**Fig. 2:**
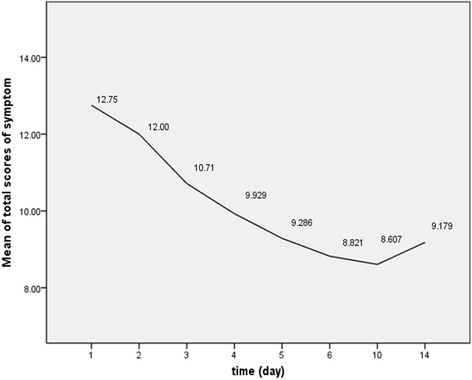
Means of total scores of all symptoms in control group on each follow-up day. The means changed statistically over time (*p* < 0.001) and decreased obviously in the first 4 days (*p* = 0.017). However, from then on no significant differences showed from day4 to day14 showed (*p* = 0.602)

Outcomes from the group C showed that the total score of all symptoms spontaneously decreased in the first 4 days (from 12.75 ± 3.52 to 9.93 ± 3.64, *p* = 0.017). However after that, no significant differences showed from day4 to day14 (from 9.93 ± 3.64 to 9.18 ± 3.38, *p* = 0.602), and a trend of increase was noted after day6 (Fig. 2).

Though the degree of symptoms changed over time, score of quality of life stayed relatively steady throughout the follow-up (*p* = 0.674) and the minimal score was 3.25 ± 1.08 (score3 means mostly satisfied while score4 means about equally satisfied and dissatisfied) (Additional file 1: Figure S1).

### Effect of endoscopic procedure on SRSs

Generally no significant differences were found in total scores of symptoms between pre- and post-lithotripsy cases (*p* = 0.066). However, subsection analysis showed that pre-lithotripsy cases had lower scores than those of post-lithotripsy ones before day4 (*p* = 0.001, mean difference = −1.455, 95% CI = −2.334 to −0.576). Subgroup analysis of single symptom suggested that within the first 4 days following insertion, pre-lithotripsy cases had milder bladder area pain (*p* = 0.036, mean difference = −0.39, 95% CI = −0.75 to −0.03), flank pain (*p* = 0.005, mean difference = −0.60, 95% CI = −1.01 to −0.19) and hematuria (*p* = 0.001, mean difference = −0.065, 95% CI = −0.34 to −0.09) comparing to post-lithotripsy cases. Pre- and post-lithotripsy cases had similar level of frequency (*p* = 0.232) and urgency (*p* = 0.825) from the beginning to the end (Additional file 1: Figure S2).

### Efficacy outcomes of medication therapy

**Fig. 3:**
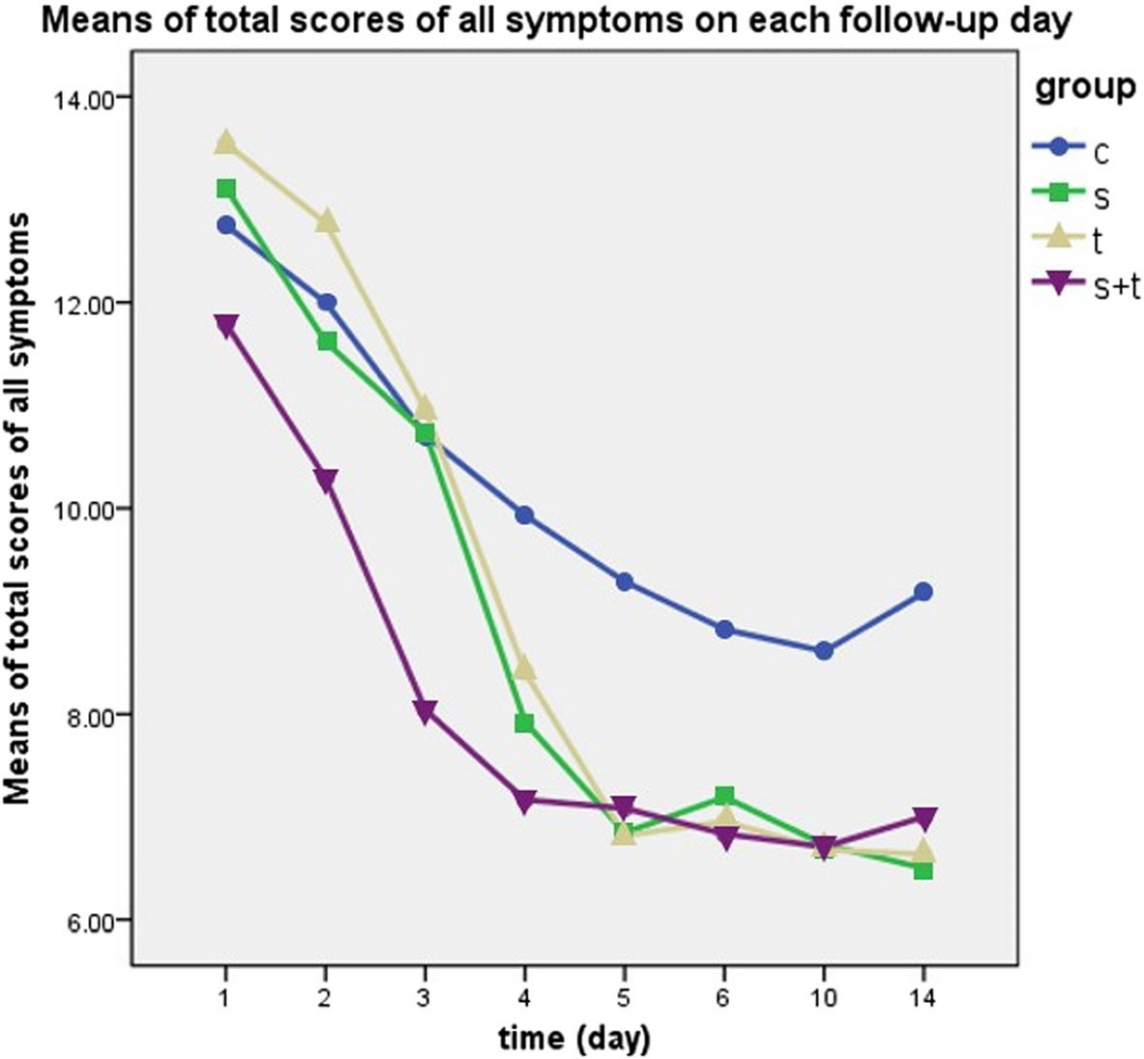
Solifenacin, tamsulosin and combination therapy all released SRSs comparing to the control group (*p* = 0.004 vs 0.026 vs <0.001). Combination therapy could release the SRSs much faster than solifenacin (*p* = 0.016) or tamsulosin (0.002) in the first 4 days. No significant differences showed up between combination and solifenacin (*p* = 0.842) or tamsulosin (*p* = 0.774) alone from day5 to day14. Solifenacin and tamsulosin showed comparable effect throughout the whole follow up (*p* = 0.582)

Overall, solifenacin, tamsulosin and combination therapy group all had lower levels of SRSs than the control group throughout the follow up (*p* = 0.004 & 0.026 & <0.001). Before day4, combination therapy provided even lower scores of SRSs than single solifenacin (*p* = 0.016, mean difference = −1.52, 95% CI = −2.76 to −0.29) and tamsulosin (*p* = 0.002, mean difference = −2.10, 95% CI = −3.39 to −0.81), but no significant differences showed up between combination and either single drug group after day5 (solifenacin & tamsulosin, *p* = 0.84 & 0.77). Solifenacin and tamsulosin showed comparable effect throughout the whole follow up (*p* = 0.582) (Fig. 3).

**Fig. 4:**
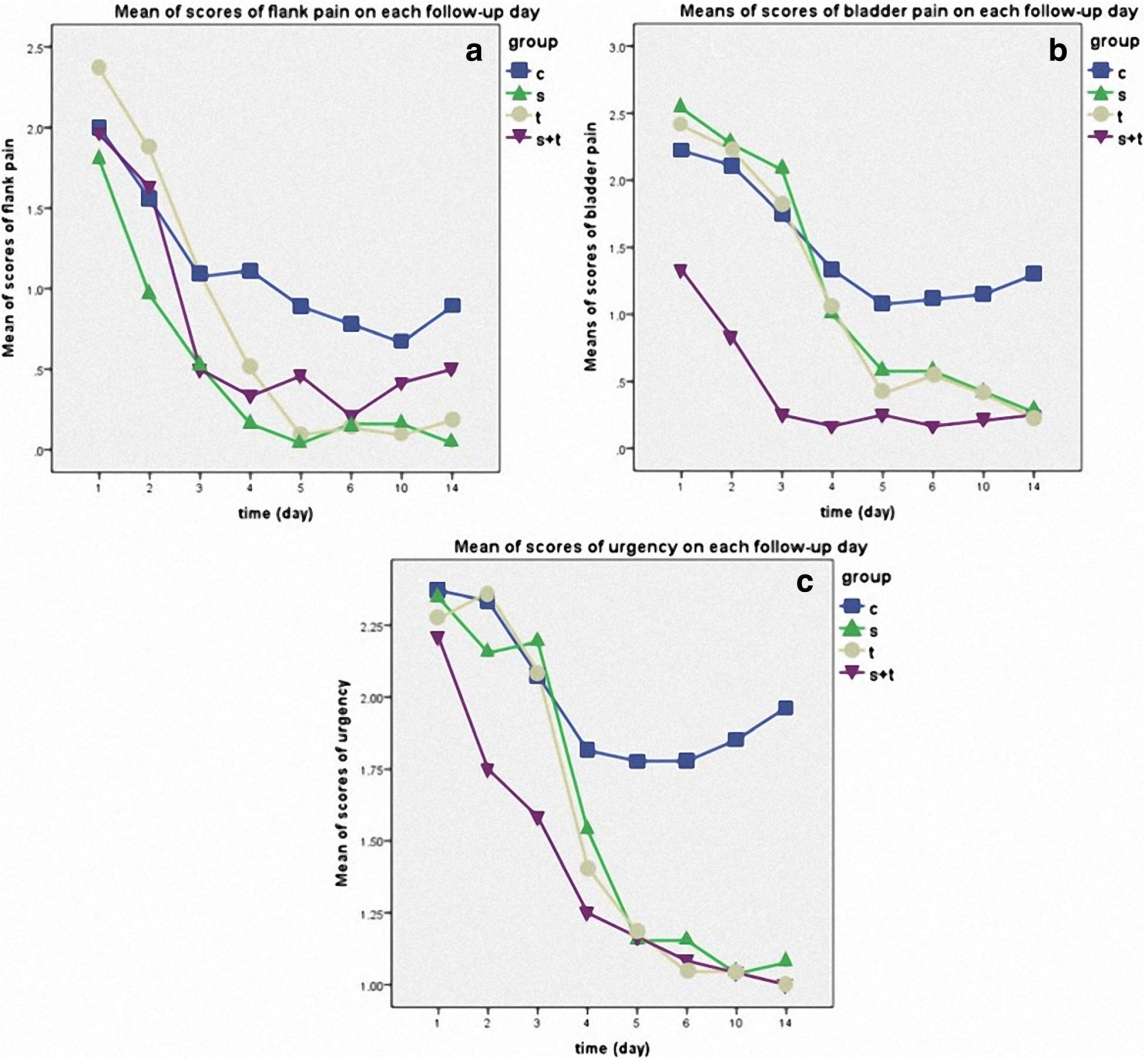
Means of scores of flank pain (**a**), bladder pain (**b**) and urgency (**c**). Effect on flank pain started showing up from day5 to the end (*p* = 0.006) and solifenacin is comparable to tamsulosin and combination therapy (*p* = 0.914 vs 0.195) (**a**). Combination therapy released bladder pain and urgency throughout the whole follow-up (comparing with the control, both *p* < 0.001) (**b** and **c**). Before day4, Solifenacin and tamsulosin had no significant effect on bladder pain (*p* = 0.589 vs 0.936) or urgency (*p* = 0.806 vs 0.729) but both showed showed comparable effectiveness as the combination therapy from day5 to day14 (**b** and **c**)

As for specific symptoms, no statistical differences were found in flank pain scores among all 4 groups before day4 (*p* = 0.101). However, from day5 to the end, a superiority over the control group was noted in solifenacin group (*p* = 0.002, mean difference = −0.71, 95% CI = −1.14 to −0.27), which was comparable with tamsulosin and combination therapy (*p* = 0.914 vs 0.195). Combination therapy released bladder pain and urgency from the very beginning and remained effective to the end (comparing with the control, bladder pain *p* < 0.001, mean difference = −1.07, 95% CI = −1.43 to −0.72; urgency *p* < 0.001, mean difference = −0.61, 95% CI = −0.91 to −0.31). On the other hand, neither of solifenacin or tamsulosin showed significant effects on bladder pain (vs control, *p* = 0.589 & 0.936) or urgency (vs control, *p* = 0.806 & 0.729) before day4. But from the fifth day on, solifenacin and tamsulosin monotherapy both started showing equal benefic effects as the combination therapy on both bladder pain (*p* = 0.229 & 0.394) and urgency (*p* = 0.813 & 0.974) (Fig. 4).

Solifenacin, tamsulosin and combination group all showed no superiority over the control group in hematuria (*p* = 0.736 & 0.924 & 1.000) or frequency (*p* = 0.073 & 0.860 & 0.092) (Additional file 1: Figure S3). Incontinence was observed on only two follow-up days from one single case in solifenacin group.

As for quality of life (QoL), significant difference existed among the 4 groups (*p* = 0.046) but combination therapy wasn’t superior to either monotherapy group (solifenacin and tamsulosin, *p* = 0.107 vs 0.670). Medication therapy groups had higher scores than the control at the beginning but finally went down to be lower after day4 (Additional file 1: Figure S4).

### Adverse events

Main complications of drug therapy groups were as followed: solifenacin group with three patients with dry mouth (3/26, 11.5%); tamsulosin group with two with dizziness (2/22, 9.1%), combination group with three with dry mouth and one with both symptoms in group (4/24, 16.7%). The total incidence rate of adverse events from these three groups and no significant differences (*p* = 0.727). No serious adverse events were reported throughout the study.

## Discussion

In a previous study on SRSs features, J. Irani et al. [12] declared that the general tolerance to SRSs remains unchanged with time while only some symptoms significantly improve, dysuria and hematuria included. And in the current study, we also recorded that though SRSs relieved spontaneously to some degree within a few days after the insertion, it would stay relatively stable after then, and might even relapse or worsen as time went by. The minimal total symptom score was designed as 4, which meant suffering no SRSs, in our questionnaire. But the actual minimal mean of total scores of all symptoms in the control group was 8.607, which again demonstrated that patients would not develop complete tolerance to SRSs within two weeks. We estimate that the symptoms appearing within the very first days after insertion may be also associated with stimulation of transurethral endoscopic procedures, and these parts of symptoms can rapidly improve. The phenomenon that pre- and post-lithotripsy cases suffered differently in the beginning may support this hypothesis to some degree. And since the patients suffer the most in the first few days, we recommend that active managements should be given to patient right after stent-insertion, especially to whom following ureteroscopy surgeries.

Speaking of efficacy, solifenacin and tamsulosin showed comparably promising effect on releasing urgency, bladder discomfort and flank pain. And the long-term effects of both monotherapies were not inferior to the combination therapy in the current study. Meanwhile however, we also noticed that in the first few days a combination therapy would take effect faster than monotherapy, especially on symptoms of bladder pain and urgency. We think that the inhibitors of α- receptor and m-receptor may have synergistic effect on releasing irritative symptoms of bladder. So for patients who have relatively severe SRSs from the beginning or who are urge to release the symptom, a combination therapy is recommended. But after the first few days, an alternation to monotherapy would be a proper choice because the outcome demonstrated that a long-term combination therapy was unnecessary.

Although some transient relieving was observed, no general improvement in hematuria and frequency was found in any medication therapy groups comparing with the control, which disagreed with some previous studies [13–15]. Hematuria is believed to result from mechanical injury on mucosa by stent, so we think it may be more likely to be affected by patients’ living habits, exercise habits for example, other than medicine intervention. And for frequency, we believe water-drinking amount also contributes a lot to it apart from stent insertion. In the current study, a daily water intake over 2500 ml resulted in urine volume increasing to about 2000 ml per day. Medication of α-blockers and anticholinergic agents are believed able to release irritating-induced storage symptoms while not affect the normal voiding function of bladder [16, 17], so frequency resulting from increased urine volume wouldn’t be improved by solifenacin or tamsulosin. This reminds us that recording daily urine volume may be necessary for an accurate SRSs assessment.

We found it interesting to note that QoL might not improve completely with symptom releasing. On the days just following stent-insertion, patients accepting drug therapies had even poorer score on QoL than the control, although the degrees of their symptoms were about the same or even better. Not satisfied with the slow effect of drug in the first few days may be one of the reasons. Also our advice on water drinking and exercise limitation, which may contribute to the lower incidence of hematuria and flank pain, may also make patients feel bothered. During the follow-up days some patients complained about change in living habits and their QoL scores stayed low even though they have no obvious symptoms.

There are three previous studies adopting the same or similar regimen with the current study. Essam S. et al. found combined therapy of 0.4 mg tamsulosin and 10 mg solifenacin daily significantly alleviated irritative symptoms associated with stent-insertion comparing to either single medication [18]. Lim KT. et al. drew a conclusion of agreement with Essam S. with half the dose [19]. Jinsung P. et al. adopted the same regimen of Lim KT., but resulted in a totally different conclusion. They declared that neither tamsulosin nor solifenacin medications provide beneficial effects for SRSs [8]. In the current study, we conducted a multiple follow-up on several different days to explore SRSs, which can avoid bias of single-day follow-up adopted by the previous studies since SRSs may be affected by aspects like amount of exercise and water-drinking. All the researches mentioned above reached only one agreement that the administration of solifenacin and tamsulosin as well as their combination appeared equally safe and no severe complications were recorded. And so did the current one.

The following limitations should be acknowledged. A major one was that our method inevitably increased the workload of follow-up staffs and participants, so a limitation existed in comprehensiveness of symptom assessment and sample size. However since the size reached our established goal, we still believe our outcomes can make some sense. Another problem was that our center only provided stents with the same size and couldn’t adjust the lengths of stents with heights. But while the randomized groups had no significant difference in patients’ heights, this limitation would have little influence on the comparison outcomes. Further studies may take more comprehensive symptoms and effects of living habits into account, ending in more accurate assessment of SRSs, so as to bring out a more optimal protocol which can benefit patients the most.

## Conclusion

As our outcomes demonstrated, SRSs would release spontaneously to some degree in the first few days after the insertion, then stay non-improved or even worsen in the following days, which may still be troublesome. Combination of solifenacin and tamsulosin can take effect faster and improve the SRSs better than monotherapy in the first few days. After that, combination and monotherapy relieve the SRSs equally. So for long-term using, patients with SRSs may get comparable benefits from monotherapy and combination. Patients with frequency or hematuria may benefit little from both drugs because these symptoms would be largely affected by living habits. Further studies with lager sample size are expected to collect more detailed data and drawing more accurate conclusions.

## Additional file

Additional file 1: Figure S1. Means of total scores of quality of life on each follow-up day. No significant difference existed from day1 to day14 (*p* = 0.674). **Figure S2**. Means of Symptom scores of pre- and post-lithotripsy cases. Generally pre- and post-lithotripsy cases had no significant differences in total scores of all symptoms (*p* = 0.066) but subsection analysis showed significant difference existed before day4 (*p* = 0.001) (a). Subgroup analysis demonstrated difference in scores of bladder area pain (*p* = 0.036), kidney area pain (*p* = 0.005) and hematuria (*p* = 0.001) (b, c, d). No obvious difference showed up on frequency (*p* = 0.232) and urgency (*p* = 0.825) from the beginning to the end. (e, f). **Figure S3**. Solifenacin, tamsulosin and combination group showed no superiority over the control group on hematuria (*p* = 0.736 vs 0.924 vs 1.000) (a). Solifenacin, tamsulosin and combination therapy didn’t effectively release the level of frequency (*p* = 0.073 vs 0.860 vs 0.092) (b). **Figure S4**. Mean of scores of quality of life. Significant difference existed among the 4 groups (*p* = 0.046) but combination therapy wasn’t superior to either single drug group (solifenacin and tamsulosin, *p* = 0.107 vs 0.670). (DOCX 807 kb)
