# Supplementary material for: Combination therapy only shows short-term superiority over monotherapy on ureteral stent-related symptoms – outcome from a randomized controlled trial
Source: BMC Urol. 2016 Nov 15;16:66. doi: 10.1186/s12894-016-0186-y (PMC5111197; doi:10.1186/s12894-016-0186-y)
Supplement: Additional file 1: Figure S1. — Means of total scores of quality of life on each follow-up day. No significant difference existed from day1 to day14 (p = 0.674). Figure S2. Means of Symptom scores of pre- and post-lithotripsy cases. Generally pre- and post-lithotripsy cases had no significant differences in total scores of all symptoms (p = 0.066) but subsection analysis showed significant difference existed before day4 (p = 0.001) (a). Subgroup analysis demonstrated difference in scores of bladder area pain (p = 0.036), kidney area pain (p = 0.005) and hematuria (p = 0.001) (b, c, d). No obvious difference showed up on frequency (p = 0.232) and urgency (p = 0.825) from the beginning to the end. (e, f). Figure S3. Solifenacin, tamsulosin and combination group showed no superiority over the control group on hematuria (p = 0.736 vs 0.924 vs 1.000) (a). Solifenacin, tamsulosin and combination therapy didn’t effectively release the level of frequency (p = 0.073 vs 0.860 vs 0.092) (b). Figure S4. Mean of scores of quality of life. Significant difference existed among the 4 groups (p = 0.046) but combination therapy wasn’t superior to either single drug group (solifenacin and tamsulosin, p = 0.107 vs 0.670). (DOCX 807 kb) [file 12894_2016_186_MOESM1_ESM.docx]

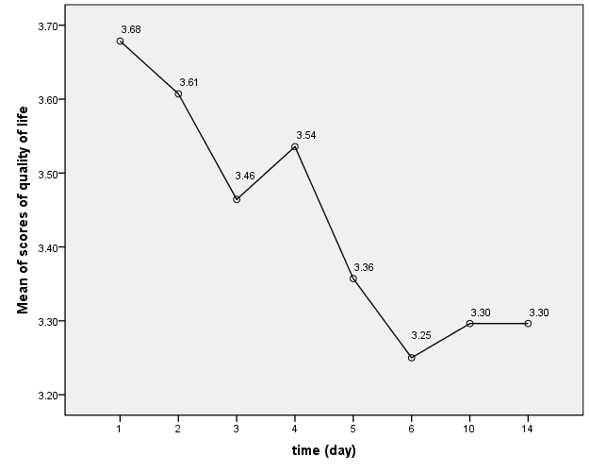


**Supplementary Figure 1** Means of total scores of quality of life on each follow-up day. No significant difference existed from day1 to day14 (p=0.674)


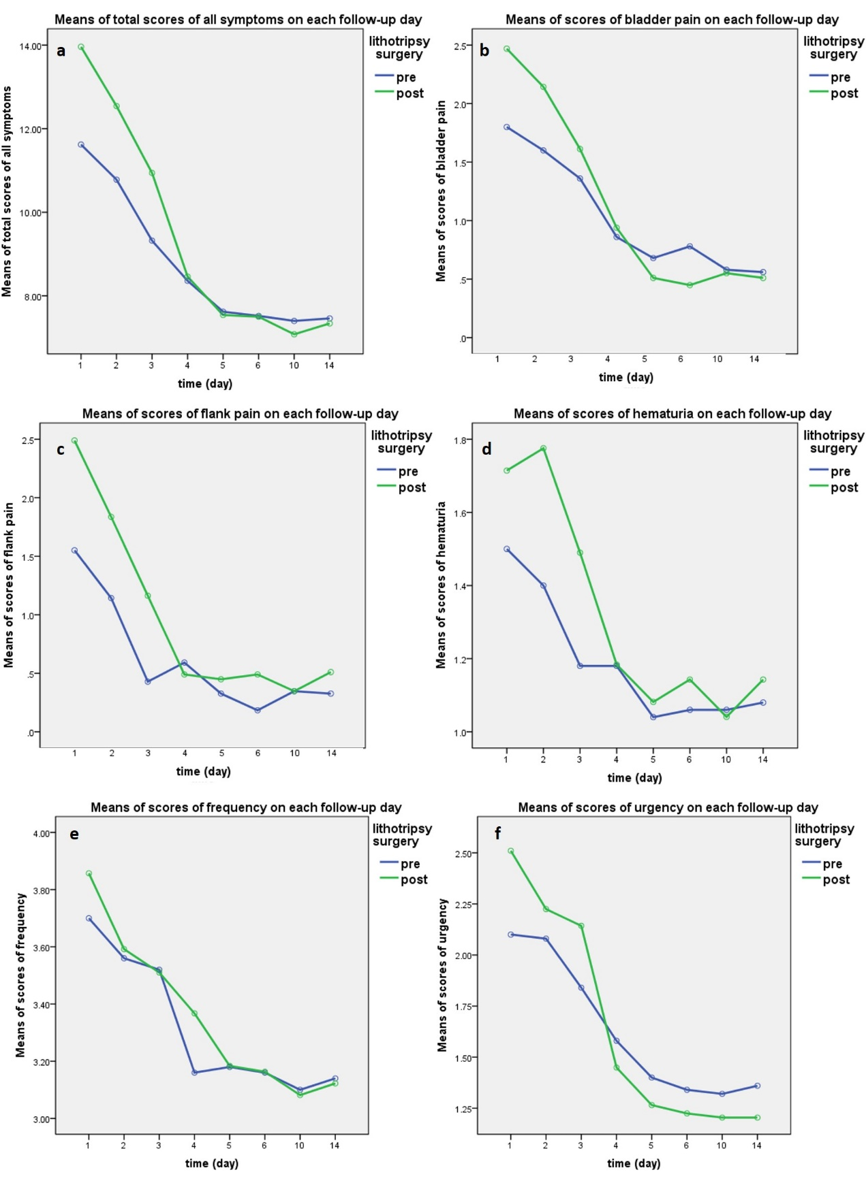


**Supplementary Figure 2** Means of Symptom scores of pre- and post-lithotripsy cases. Generally pre- and post-lithotripsy cases had no significant differences in total scores of all symptoms (p=0.066) but subsection analysis showed significant difference existed before day4 (p=0.001) (a). Subgroup analysis demonstrated difference in scores of bladder area pain (p=0.036), kidney area pain (p=0.005) and hematuria (p=0.001) (b, c, d). No obvious difference showed up on frequency (p=0.232) and urgency (p=0.825) from the beginning to the end. (e, f)

**Supplementary Figure 3** Solifenacin, tamsulosin and combination group showed no superiority over the control group on hematuria (p=0.736 vs 0.924 vs 1.000) (a). Solifenacin, tamsulosin and combination therapy didn’t effectively release the level of frequency (p=0.073 vs 0.860 vs 0.092) (b).


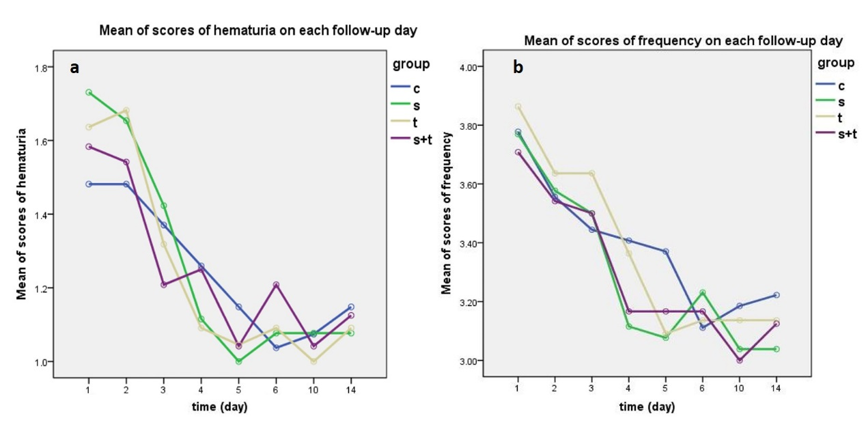

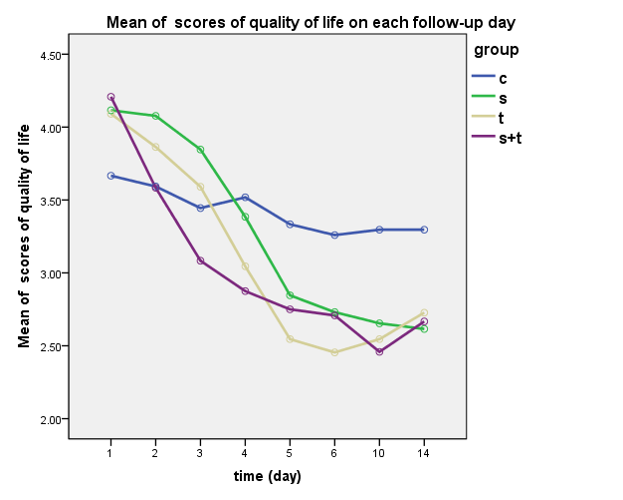


**Supplementary Figure 4** Mean of scores of quality of life. Significant difference existed among the 4 groups (p=0.046) but combination therapy wasn’t superior to either single drug group (solifenacin and tamsulosin, p=0.107 vs 0.670).
